# Injection of autologous conditioned plasma combined with a collagen scaffold may improve the clinical outcome in shoulder impingement syndrome: a prospective study

**DOI:** 10.1007/s00590-023-03595-x

**Published:** 2023-05-30

**Authors:** Agnieszka Halm-Pozniak, Christoph H. Lohmann, Friedemann Awiszus, Margit Rudolf, Jessica Bertrand, Alexander Berth

**Affiliations:** Department of Orthopaedic Surgery, Otto-Von-Guericke-University Magdeburg, 44 Leipziger St., 39120 Magdeburg, Germany

**Keywords:** Shoulder impingement syndrome, Autologous conditioned plasma, Collagen scaffold, Growth factors

## Abstract

**Background:**

Shoulder impingement syndrome (SIS) is one of the most common diseases of the shoulder and can be addressed with various therapeutic concepts. Orthobiological agents such as platelet rich plasma with a low side effect rate gain importance in the conservative treatment of SIS. Currently, the knowledge about success rate influencing factors, such as the growth factors (GF) concentration or acromion type, is limited. The aim of this study was to analyze the clinical outcome in the therapy of external SIS using autologous conditioned plasma combined with recombinant human collagen scaffold (ACP/STR) injection in comparison with a corticosteroid-local anesthetic (CSA) injection. Additionally, the influence of potential limiting factors such as GF concentration, age and acromial morphology was proved.

**Materials and methods:**

This prospective pseudo-randomized trial recruited 58 patients with external SIS who received an ultrasound-guided subacromial injection either an ACP/STR or a CSA followed by physical therapy. Follow-up (FU) was performed at 6 weeks, 3 and 6 months. The outcome was assessed with Constant–Murley score, disability of arm, shoulder and hand score and simple shoulder test. The concentration of GF was measured using ELISA.

**Results:**

During the FU, the improvement of outcome measures was observed with no differences between both groups. Shoulder force was significantly increased in the ACP/STR group (*p* < 0.01). We found no correlation between the amount of GF and age or gender in the ACP/STR patients. An acromion Bigliani type III predisposes for therapy failure (*p* < 0.001, OR = 56) in both treatment groups.

**Conclusions:**

Patients with SIS benefit regarding to PROMs after both ACP/STR and CSA injection and physical therapy. Patients who received ACP/STR obtained superior improvement in force. The quantity of GF did not vary depending on the age, so that ACP/STR can be a treatment option for SIS in elderly patients with multimorbidity. The presence of an acromion type III seems to be a predictive factor for limited effectivity of injections in the clinical management of SIS.

## Introduction

Shoulder impingement syndrome (SIS) has a prevalence of 10–16% in the general population and is one of the most common musculoskeletal diseases. If not treated, it may lead to disability of the arm and, as a consequence, to reduced quality of life [1]. For this reason, it is important to properly diagnose the disease and choose the appropriate therapy for each patient.

SIS therapy is a symptom-focused intervention. First of all, the non-surgical options should be addressed. There are two major complains by SIS-pain and functional disability. The first one, pain, can be treated by oral analgesics and physiotherapy. If it is not sufficient, the next step is an infiltration of the subacromial space either with local anesthesia, cortison or both. The common use of combined corticosteroid-local anesthetic (CSA) injections in SIS is controversially discussed in the current literature [2]. A rapid pain relief is to be expected after CSA injection; however, the long-lasting healing effect on the tendon may not be achieved. Other therapeutic options including orthobiologics such platelet rich plasma derivatives (e.g., autologous conditioned plasma—ACP) continue to be used at increasing rates in the treatment of musculoskeletal disorders [3, 4]. According to other studies, pain and range of motion may show similar or higher improvement with the use of PRP [5]. Various studies have reported a wide spectrum of ACP use in orthopedics, including wound healing, support of fracture or nonunion repair and tendon pathologies, or epicondylitis [6–9]. The content of many growth factors in ACP may support the conditions for better tendon healing and regeneration due to enhanced activation of cytokines, vascularization and collagen production [4, 10–13]. Although all GF play a major role in the anti-inflammatory reaction, PDFG, TGF ß, HGF and EGF seem to have major influence on the tendon tissue [14].

The potential advantages of a combination of ACP and a plant scaffold with recombinant human collagen type I (ACP/STR) has not yet been investigated until now. One single injection of the ACP/STR is hypothesized to be less traumatizing than the standard of multiple injections of ACP or repeated CSA injections with a decreased risk for complications. Moreover, the scaffold is expected to provide longer retention and bioavailability of the GFs in subacromial space, thereby preventing an early wash-out of GFs from the injection site [11, 12, 15, 16].

Therefore, we questioned if non-operative treatment of SIS due to rotator cuff tendinopathy (RCT) or partial-thickness rotator cuff tears (PTRC) with a single subacromial ACP/STR injection, and physical therapy may be superior to CSA injection and physical therapy. Additionally, we proved the influence of potential limiting factors such as GF concentration, age and acromial morphology.

## Materials and methods

### Patients cohorts

Fifty-eight patients with external outlet and non-outlet SIS were recruited for our study and randomly divided into two groups of 29 patients. We performed a pseudo-randomization. Patients with an even checking number became ACP/STR injection, and those with uneven checking number became the CSA injection. Patients were enrolled from February 2018 to July 2020 in the outpatient clinic of the Department of Orthopedics Surgery. Informed written consent was obtained from all patients prior to inclusion in the study. The study was approved by the Institutional Review Board.

The inclusion criteria for the patients were SIS symptoms for less than 6 months, age > 18 years, no invasive/surgical therapy before, radiological-confirmed presentation of SIS caused by rotator cuff tendinopathy (RCT) or partial-thickness rotator cuff tears (PTRC) Ellman type I-II [17] and subacromial bursitis with or without extrinsic impingement from acromial spur or osteophytes on acromioclavicular joint. The exclusion criteria were full-thickness RC tears, osteoarthritis of the shoulder, systemic inflammatory or neuromuscular diseases, age < 18, SIS therapy in the last 6 weeks, previous subacromial injections, surgery for SIS and general contraindications for this therapy.

**Fig. 1:**
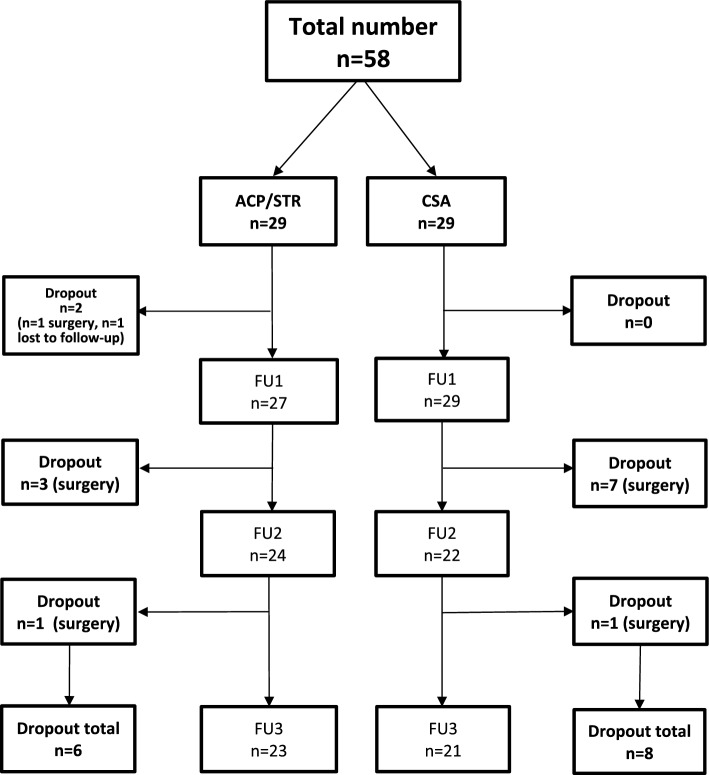
Flowchart study group recruitment

The descriptive data of the patients are summarized in Table 1. 27 male and 31 female patients were included. The mean age was 51.9 (range: 26–79) years in the total cohort. 6 patients dropped out of the study (*n* = 5 conversion to surgery, *n* = 1 lost to FU) in the ACP/STR group and 8 patients (*n* = 8 conversion to the surgery) dropped out in the CSA group. The final number of patients was 44 (Fig. 1).

**Table 1:** Patient data according to the group

	ACP/STR (X̅ ± SD)	CSA (X̅ ± SD)
Age (years)	52.17 ± 12.43	51.62 ± 11.67
Minimum	28	29
Maximum	78	79
Gender (f/m)	16/13	15/14
Side (r/l)	16/13	15/14
Follow-up 1 (days)	51.98 ± 10.54	45.89 ± 14.24
	(37–74)	(31–68)
Follow-up 2 (days)	101.78 ± 16.50	97.93 ± 18.80
	(73–142)	(75–135)
Follow-up 3 (days)	206.49 ± 25.19	186.57 ± 30.00*
	(170–248)	(143–225)
*Acromion type*		
I	2 (6.9%)	4 (13.8%)
II	22 (75.9%)	20 (69.0%)
III	5 (17.2%)	5 (17.2%)
*RC pathology*		
RCT	13 (44.8%)	15 (51.7%)
PTRC	9 (31.1%)	9 (31.1%)
Fatty degeneration	7 (24.1%)	5 (17.2%)

(X̅ ± SD) mean ± standard deviation

*RC* rotator cuff, *RCT* rotator cuff tendinopathy, *PTRC* partial-thickness rotator cuff tear, *ACP/STR* autologous conditioned plasma with recombinant human collagen scaffold, *CSA* corticosteroid-local anesthetic

*p = 0.02

### Radiological assessment

Standardized radiographs (true anteroposterior, outlet and axillary view) were evaluated for the presence of bony changes and to assess the acromion morphology according to Bigliani [18]. MRI-scans (T2- and PD-weighted sequences) in coronary, parasagittal and axial plane were evaluated for the status of the rotator cuff. RCT was defined as only intensity changes in the rotator cuff and absence of disruptions in the tendon. PTRC was defined as a bursal, articular or intratendinous tendon disruption that did not involve the entire thickness of the tendon.

### Procedures and interventions

For the ACP/STR preparation, we used a double lumen syringe (Arthrex ACP™ Double Syringe, Arthrex Inc. Naples, FL, USA) and 15 ml blood were taken from the patients followed by 5 min of centrifugation (1500 turns/min, Rotofix 32A, Hettich). The obtained plasm a was immediately mixed with the collagen scaffold Vergenix STR, an engineered human collagen extracted from tobacco plants (STR, CollPlant Ltd., Ness-Ziona, Israel) and subsequently applied into the subacromial space [19, 20].

The CSA group received 10 ml bupivacaine (0, 5%) and 2 ml of dexamethasone.

The injections for both groups were performed under sterile conditions, as well as ultrasound guided, from the lateral aspect of the proximal humerus into the subacromial space.

All injections were performed by the same clinician. After injection, all patients underwent a standard rehabilitation protocol by the same physical therapist.

In the first two weeks, the patients received manual therapy for the shoulder including cervical spine, ergotherapy and manual lymphatic drainage as well as exercises in the pain-free range of motion. In the further 3–6 weeks, the therapy was continued with arm muscle stimulation/building and extension of ROM against resistance.

### Analysis of growth factor content

1–2 ml of the prepared plasma was separately placed into a tube with ethylenediaminetetraacetic acid and immediately frozen at − 80° Celsius. The mean storage time was about 236. 9 ± 66. 8 days (23–304 days). Samples were thawed at the same time for subsequent ELISA (enzyme linked immunosorbent assay) testing. We examined the concentration of the following GFs using the following ELISA Kits: human HGF #DHG00, human TGF beta 1 #DB100B, human EGF #DEG00, human PDGF #DHD00C (R&D, Minneapolis, USA). All ELISA were performed according to the manufacturer’s instructions. All samples were measured in duplicates and the mean value was calculated.

### Outcome measurements

The patients were examined using the Constant–Murley score (CS) [21] the disability of arm, shoulder and hand score (DASH) [22] and the simple shoulder test (SST) [23] for the assessment of shoulder function and mobility. This evaluation was performed prior to the injections and at 6 weeks, 3 months and 6 months after the intervention.

### Statistical analysis

The statistical analysis was performed with SPSS version 27 for Windows. A priori power analysis (G*Power Version 3.1.2 software) indicated that 58 patients will be needed to detect a clinically relevant effect size (0.5) with a power of 80%. The results are presented as mean values ± standard deviation, unless otherwise stated. A *p* value < 0.05 was considered significant. The unpaired t test was used to show significant differences between both study groups. We used repeated measures multifactor analysis of variance with intra subject factor time (FU 1, 2, 3) and inter subject factor intervention (ACP/STR vs. CSA). When necessary, a post hoc test for the least significant differences was performed. The Pearson correlation coefficient was calculated to measure the correlation between individual GF concentration and patient’s age. The Chi squared test was used for analyzing the influence of acromion morphology on the clinical response to the intervention.

## Results

### Patients

There were no significant differences between the groups with regard to age (*p* = 0.86), gender (*p* = 0.79) and follow-up 1 and 2 (*p* = 0.07 and *p* = 0.45), whereas follow-up 3 differs between the groups (*p* = 0.02) (Table 1). The preoperative functional status of the patients in both groups is represented in Table 2, which shows no differences.

**Table 2:** Baseline and follow-up values of constant score, DASH score and SST in the entire series

	Baseline	Follow-up 1	Follow-up 2	Follow-up 3	*F* value	*p* value
*Total adj. (points)*						
ACP/STR	70.1 ± 23.3	86.4 ± 20.3	95.6 ± 18.6	96.9 ± 21.5	1.19	0.30
CSA	63.5 ± 18.5	75.0 ± 23.2	81.8 ± 21.2	85.0 ± 24.6		
*Pain (points)*						
ACP/STR	8.1 ± 4.5	11.2 ± 4.5	13.0 ± 3.5	13.0 ± 3.6	0.77	0.46
CSA	7.7 ± 2.8	9.8 ± 3.8	11.1 ± 4.0	11.6 ± 3.9		
*Activity (points)*						
ACP/STR	12.2 ± 4.9	14.9 ± 4.6	16.0 ± 4.8	16.4 ± 4.7	0.75	0.46
CSA	10.3 ± 4.4	13.5 ± 4.9	13.9 ± 4.5	15.4 ± 4.5		
*CS motion (points)*						
ACP/STR	29.3 ± 8.6	33.2 ± 7.8	36.4 ± 5,6	36.0 ± 7.6	1.26	0.28
CSA	25.3 ± 8.5	27.3 ± 9.9	31.1 ± 8.1	30.4 ± 9.7		
*CS force (points)*						
ACP/STR	9.0 ± 4.9	13.2 ± 6.2	14.5 ± 5.9	15.3 ± 6.1	4.21	0.01^*^
CSA	9.6 ± 3.4	11.0 ± 4.2	11.3 ± 4.2	12.3 ± 4.1		
*DASH (points)*						
ACP/STR	36.0 ± 21.9	31.5 ± 19.3	24.6 ± 19.6	15.7 ± 21.4	0.79	0.44
CSA	34.2 ± 17.3	27.4 ± 12.8	25.1 ± 15.2	13.8 ± 17.7		
*SST (points)*						
ACP/STR	5.6 ± 3.1	7.4 ± 3.4	8.3 ± 3.1	8.5 ± 3.5	0.15	0.84
CSA	6.4 ± 2.6	8.1 ± 2.4	8.4 ± 2.4	8.7 ± 2.8		

^*^ Relevant improvement

*ACP* autologous conditioned plasma with recombinant human collagen scaffold; *CSA* corticosteroid-local anesthetic

### Clinical results

**Fig. 2:**
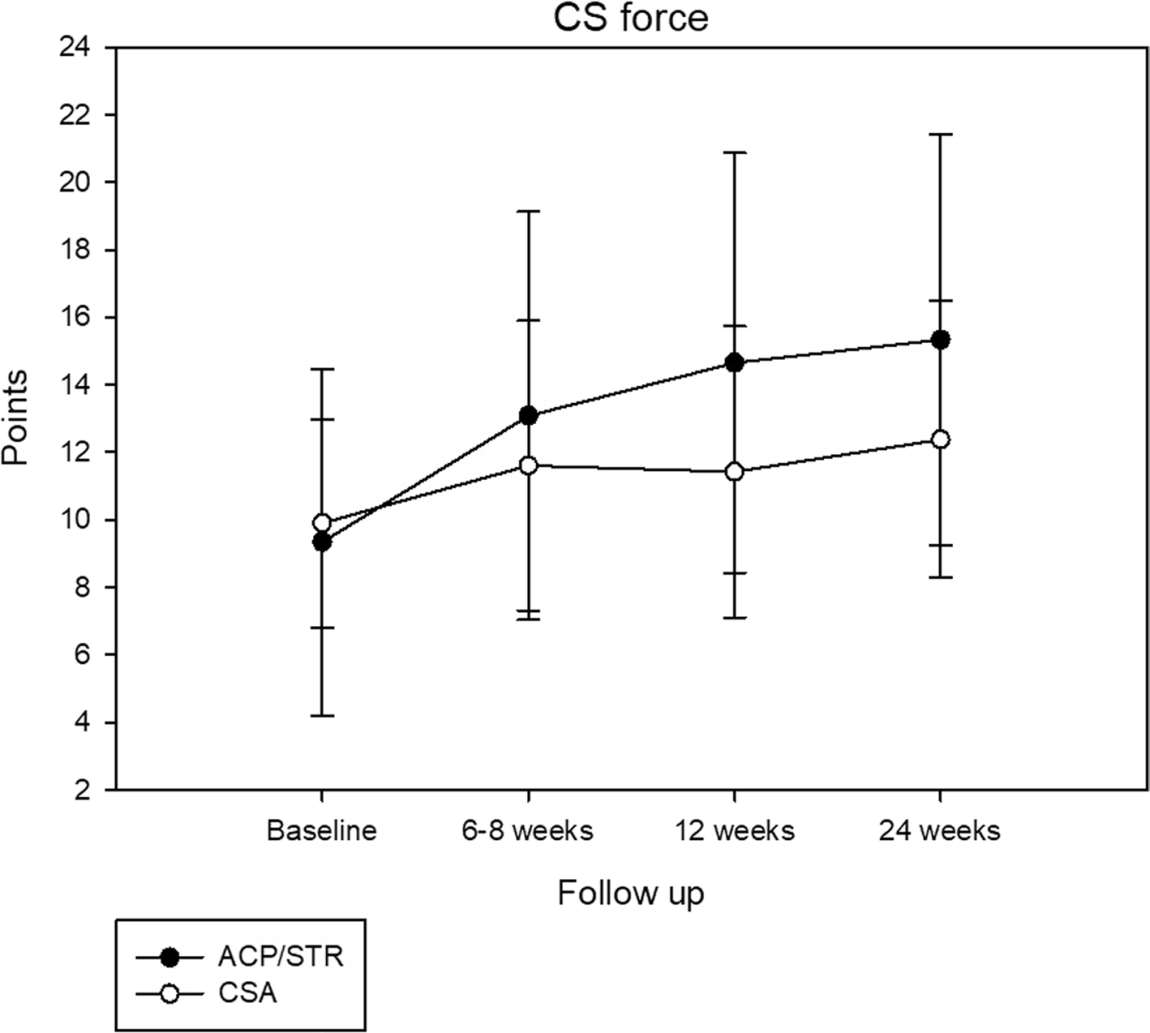
Constant score force

The clinical results during the FU demonstrated significant improvements compared with the baseline values in all patients reported outcome measures for both patient groups. There were no significant differences between the groups with respect to the CS Score (*p* = 0.30), DASH score (*p* = 0.44) and SST (*p* = 0.84) apart from force in CS Score which was increased in the ACP/STR group (*p* = 0.01, Table 2, Fig. 2) compared to the CSA group.

### Complications

There were no complications in both groups. 70% of the patients complained about increase in pain directly after obtaining the ACP/STR Injection that resolved after 4 h.

### Growth factor analysis

The quantitative analysis of GF in ACP revealed that the respective concentration was not dependent on age (Pearson-test, EGF *r* = 0.043, *p* = 0.83; TGF-β1 *r* = 0.169, *p* = 0.39; PDGF *r* = 0.097, *p* = 0.63; HGF *r* = 0.033, *p* = 0.86) or gender (Pearson-test, EGF *r* = 0.26, *p* = 0.18; TGF-β1 *r* = 0.25 *p* = 0.19; PDGF *r* = 0.22, *p* = 0.26; HGF *r* = 0.04, *p* = 0.85). EGF reached the highest concentration with 531.81 ± 175.0 pg/ml, followed by TGF-β1 with 61.72 ± 41.8 pg/ml, PDGF with 35.85 ± 12.2 pg/ml and HGF with the lowest concentration of 24.39 ± 7.9 pg/ml.

### Radiological evaluation

Rotator cuff pathologies and the distribution of acromion type are assessed (Table 1).

In the ACP/STR group, all patients who had acromion type III (*n* = 5) discontinued therapy due to persistent symptoms within the FU period. In the CSA group, this was observed in 4 out of 5 patients who had acromion type III. Therefore, it could be shown that an acromion Bigliani type III predisposes for therapy failure (*p* < 0.000, OR = 56), independently from the used medication for the injection.

## Discussion

To our knowledge, this is the first prospective study to assess the effects of a scaffold-based ACP formulation compared with CSA administered with ultrasound-guided subacromial injections as therapy of SIS. The results present clearly that autologous GF applications are at least as effective, with even greater improvement of the force score, as the injections of CSA. The GF content does not depend on the age and gender of the patients.

Our study confirmed that ACP/STR has a therapeutic effect on patients with SIS diagnosis. Combined with a physiotherapeutic counseling, we achieved 79.31% of positive outcome in both pain relief and improvement of functionality. Similar to 72.41% of positive outcome in the second group, suggesting that ACP/STR is at least as beneficial as the well-established therapy including steroid injections [5], but without typical side effects such as allergic reactions or tissue necrosis by steroid use [2, 24, 25]. Moreover, the ACP/STR patients reached a statistical relevant improvement of shoulder forces. Both SIS treatment methods have been proven effective independently with level I EBM established [26]. It has been proposed that the increasing evidence for the beneficial role of growth factors in reconstructive orthopedics, especially in tendon pathologies, and the easy preparation and application technique of this treatment makes the potential application in patients with SIS a viable alternative [27]. Other studies already showed ex vivo a positive effect on tendons and especially on the rotator cuff tissue [28–30]. The novel approach of this study, by combining the ACP with a collagen scaffold, may serve for a longer retention of GFs in the subacromial space and as a consequence better healing of the tendon [15, 31]. However, we investigated our patients at 6 months follow-up. Further studies are necessary to estimate the long-lasting effects. Moreover, only one injection instead of three (as commonly practiced without a scaffold) decreases the infection rate and the stress for the patients. Furthermore, this technique decreases cost by reducing outpatient clinic visits involving disposables as well as personnel. So far, there are no reports describing the combination of ACP and a collagen scaffold for SIS treatment. However, Farkash et al. used this method for elbow epicondylitis with a good functionality and strength improvement after 6 months [32]. This may be also advantageous compared to previous studies using 3 consecutive injections to the epicondyles by Tetschke et al. [9]. Unfortunately, this therapy is still quite expensive, and it is the patient who incurs the cost, as the ACP/STR has not been established yet as a standard procedure by SIS. This creates in the sociological point of view potential conflicts.

In our study, we did not observe any side effects in both cohorts. However, almost all patients in the ACP/STR group complained about an initial painful and pressure at the site of injection during 4 h post injection. This effect might be explained by the high viscosity of the mixed plasma and collagen scaffold. However, these symptoms resolved completely the day after treatment. An additional dose of local anesthetic to relief the pain sensation is not recommended, because it may stop or decrease the desired pro inflammatory tissue reaction [33]. In addition, attention should be paid to pause any non-steroidal anti-inflammatory or platelet blocking medication before starting the ACP therapy to guarantee the best possible function of thrombocytes.

The level of PDFG, TGF ß, HGF and EGF was determined as this GF can be found in the literature playing major role by tendons healing and regeneration process [14]. No correlation was found between the growth factors concentration and age of the individual patient. However, the concentration of each GF varied between the samples. Therefore, it can be assumed that it is not the very high amount of GF that matters, but the individual increased concentration of all GF that induces the repair or anti-inflammatory process. Our findings are in concordance with other studies [14, 34]. Furthermore, Dhurat et al. observed in their study that more factors than the pure GF concentration may influence the ACP beneficial effects, such as temperature and technique of centrifugation, blood sampling or anticoagulative medication [35]. However, there are also reports that the GF concentration decreases at patients aged over 60 [28]. In our study, we did not observed this effect which may account for an optimal platelet isolation and GF release for all ages with the used technique. In this regard, it can be assumed that a single ACP injection combined with a scaffold is an alternative treatment option for SIS even in elderly patients who are often additionally characterized by multimorbidity. Although SIS typically occurs at middle age, we registered also a clinical improvement by younger patients with SIS symptoms. Due to this, it can be a valid treatment option for professional athletes as this therapy is not considered illegal doping. Moreover, due to the no reported adverse events of ACP as a biological agent makes it a safe intervention in comparison with CSA injections used in the clinical management. However, the potential changes of GF concentration due to daily hormonal stimulation and eventual influence of prior sport activity have not been investigated in our study.

Moreover, this study clearly demonstrates that the acromion type is a predictor for the outcome of conservative therapy. 83% of the drop out patients in the ACP/STR group and 62.5% of the drop out patients in the control cohort exhibited an acromion type Bigliani III. Almost all of these patients underwent surgery since the conservative therapy including subacromial injection, either with CSA or ACP/STR, failed to improve the symptoms. The findings of the present study are in accordance with the study of Berná-Mestre et al., who also demonstrates that acromion type Bigliani III is a predictive of unfavorable results in SIS patients treated with platelet rich plasma injections [36]. As a consequence, acromion type Bigliani III in patients with SIS should be reconsidered as a critical factor for a future study, since the acromion type seems to an overriding variable in the treatment of SIS. However, some further casual relations such as the presence and size of RCT should be considered.

As a limitation of this study, we included only a small patient number and longer FU as 6 months could tell more about the sustainability of the ACP/STR therapy. Another methodical aspect of this study is that the final FU investigation differed significantly between the groups. However, it can be assumed that these FU difference of about 20 days do not have an essential impact of the given clinical results. Individual reasons were mainly responsible to this FU difference and the design of the present study reflecting therefore more the daily clinical practice.

## Conclusion

During the FU, patients with SIS due to RCT and PTRC benefit regarding to PROMs after both ACP/STR and CSA injection and subsequent physical therapy. Patients who received ACP/STR injection obtained superior improvement in force. The quantity of different GF did not vary depending on the age of the patient, so that ACP/STR can be initially used as a treatment for SIS in patients with multimorbidity or contraindications for CSA. The presence of an acromion type III seems to be a predictive factor for limited effectivity of subacromial ACP and CSA injections in the treatment of SIS. Further investigations with a longer FU are needed to evaluate the efficacy of ACP/STR in the management of SIS.
